# A Fully Convolutional Network-Based Tube Contour Detection Method Using Multi-Exposure Images

**DOI:** 10.3390/s21124095

**Published:** 2021-06-14

**Authors:** Xiaoqi Cheng, Junhua Sun, Fuqiang Zhou

**Affiliations:** School of Instrumentation Science and Opto-electronics Engineering, Beihang University, Beijing 100191, China; chengxiaoqi@buaa.edu.cn (X.C.); zfq@buaa.edu.cn (F.Z.)

**Keywords:** fully convolutional network, tube contour detection, multi-exposure images, U-Net, dilation operation

## Abstract

The tube contours in two-dimensional images are important cues for optical three-dimensional reconstruction. Aiming at the practical problems encountered in the application of tube contour detection under complex background, a fully convolutional network (FCN)-based tube contour detection method is proposed. Multi-exposure (ME) images are captured as the input of FCN in order to get information of tube contours in different dynamic ranges, and the U-Net type architecture is adopted by the FCN to achieve pixel-level dense classification. In addition, we propose a new loss function that can help eliminate the adverse effects caused by the positional deviation and jagged morphology of tube contour labels. Finally, we introduce a new dataset called multi-exposure tube contour dataset (METCD) and a new evaluation metric called dilate inaccuracy at optimal dataset scale (DIA-ODS) to reach an overall evaluation of our proposed method. The experimental results show that the proposed method can effectively improve the integrity and accuracy of tube contour detection in complex scenes.

## 1. Introduction

**Figure 1:**
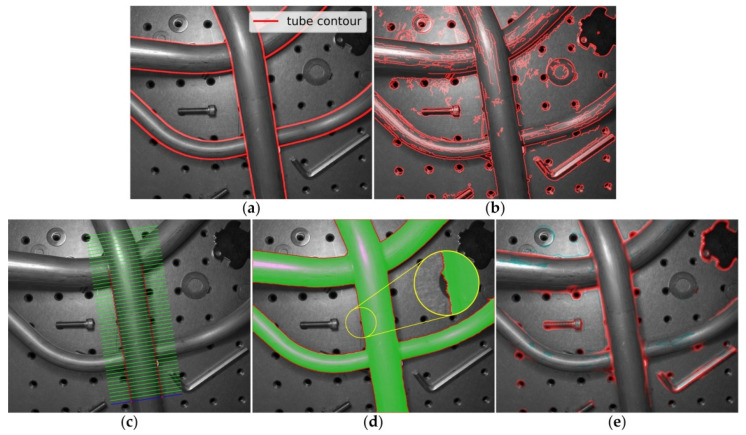
Tube contours detected by different methods. (**a**) Ground truth; (**b**) Canny [4]; (**c**) Aubry [8]; (**d**) U-Net [12]; and (**e**) HED [13].

Tubes are widely used in the fields of aerospace, automobiles, ships, and other fields for transporting liquids or gases such as fuel, coolant, and lubricating fluid. These tubes are generally metallic. The contours of tubes in two-dimensional images usually appear as edges containing certain shallow features (such as gradient, intensity) and deep features (such as texture, shape, and spatial relation), as shown in Figure 1a. Accurate detection of these contours is very important for achieving three-dimensional reconstruction and measurement of tubes [1,2,3]. In theoretical research and practical applications, many scholars have proposed various methods that can be used for the detection of tube contours.

In the field of image processing, many edge detection algorithms have been proposed [4,5], and some of them have been used to perform tube contour detection in some single tube measurement applications [3,6]. These kinds of methods only require the gradient information in the image to complete the edge detection work. Therefore, these methods have the advantage of simple design, easy operation and high efficiency. However, due to the lack of high-level features, these methods are easily disturbed by messy backgrounds, uneven lighting and ambient noise.

There are some other researchers [7,8] who adopted the combination of multiple feature descriptors, such as texture, shape and spatial relation, to realize the tubular object recognition under general background. To a certain extent, these kinds of methods improve the robustness and stability of the result. However, these methods need a considerable amount of expertise for the precise modeling of deep features, which is not only difficult to design but also limits the scope of applications.

Nowadays, deep learning technology has achieved excellent performance in various fields. Especially, fully convolutional networks (FCN) can take arbitrarily sized images as input and achieve pixel-level dense prediction, so they are widely used for image segmentation [9,10], edge detection [11] and so on. Existing FCN models, such as U-Net [12] and HED [13], can also be used for tube contour detection with a little modification. Since the training process of neural networks enables the automatic learning of feature hierarchies, these kinds of methods show great potential in the tube contour detection task. However, the existing FCN models are not designed especially for the task of tube contour detection, and the detection results commonly have problems such as low positioning accuracy and low integrity.

In the present study, aiming at the practical problems encountered in the application of tube contour detection under complex background, an FCN-based tube contour detection method is proposed. Meanwhile, we introduce a new dataset and a new evaluation metric to achieve an overall evaluation of our proposed method.

### 1.1. Related Work

In this paper, the previous works on tube contour detection are divided into three categories: gradient-based methods, multiple feature-based methods and deep learning-based methods.

#### 1.1.1. Gradient-Based Methods

In the pipeline multi-camera reconstruction system established by Zhang et al. [6], the classical Canny algorithm [4] was adopted to realize tube contour detection, while in the tube axis reconstruction method described by Sun et al. [3], an accurate sub-pixel edge location algorithm based on partial area effect [5] was used to detect tube contours. These methods require the tube contours to appear as step edges, and the edge detection processes depend heavily on the gradient features. Therefore, these methods are easily affected by messy backgrounds, and backlights are generally required in practical applications to obtain high-contrast images with clear tube contour edges. Figure 1b shows the edge detection result of Figure 1a by the Canny algorithm [4].

#### 1.1.2. Multiple Feature-Based Methods

Thirion et al. [7] proposed to implement tube segmentation in industrial images by fusing methods from physics-based vision, edge and texture analysis, probabilistic learning and the use of the graph-cut formalism. Figure 1c shows the tube contour detection result of Figure 1a by the method proposed by Aubry et al. [8]. In this method, an intensity profile is first obtained by manually defining a parallel strip segment primitive (the blue line in Figure 1c), then the tubular object recognition process will be accomplished by matching of this intensity profile (the green line in Figure 1c). The tube contour detection task can be further accomplished based on these methods. Due to the combination of multiple features, these methods have shown better performance under general background than methods that use gradient features only.

#### 1.1.3. Deep Learning-Based Methods

Here, we focus on the potential of FCN models in tube contour detection task. The idea of FCN was first introduced by Long et al. [14] for image segmentation. The biggest characteristic of this network is that it converts fully connected layers of traditional convolutional neural network to convolutional layers. Later on, Ronneberger et al. [12] proposed U-Net, which consists of a contracting path to capture context and a symmetric expanding path that enables precise localization. This network has achieved excellent performance in segmentation tasks with small dataset sizes. Such as Bardis et al. [15] proposed to use U-Net to realize organ segmentation with 8~320 samples, and Wang et al. [16] modified U-Net for ore image semantic segmentation with 76 samples. Subsequent networks such as SegNet [17], DeepLab series [18,19,20] and PSPNet [21] are all FCN-based, and they have shown good performance on segmentation tasks with large-scale datasets. The networks mentioned above can also be used for tube region segmentation directly, but further post-processing is needed to obtain tube contours. Figure 1d shows the tube segmentation result of Figure 1a by the U-Net [12]. Although the tube regions are well segmented (the green area in Figure 1d), the positioning accuracy of tube contours is poor (the red contours in Figure 1d). This is mainly because the training process of segmentation network pays more attention to the classification results of tube’s inner regions, which occupy a large proportion, rather than the classification results of contour points, which occupy a small proportion.

Other type of FCN-based networks, such as HED [13] and its variants [22], are designed for general edge detection task. The HED network inserts a side-output layer after the last convolutional layer of each stage in a VGGNet [23], and deep supervision is imposed at each side-output layer, so that the result is toward the edge detection. Figure 1e shows the edge detection result of Figure 1a by the HED [13]. Due to the lack of semantic description of the detected edges, these networks cannot be directly used for tube contour detection under complex background.

In order to detect the edges of specific objects, Yu et al. [24] defined a category-aware semantic edge detection task and proposed CASENet to complete the task. The CASENet adopts a modified ResNet-101 [25] architecture with dilated convolution as the backbone and uses a multi-label loss function to supervise the training process. In recent years, some other networks [26,27] have been proposed for the same purpose. Both of these networks have achieved excellent performance on large standard benchmark datasets such as SBD [28] and Cityscapes [29]. Nevertheless, tube contour detection under complex background is still a challenging task in practical applications, mainly due to the following two aspects:

- Poor tube image quality. For metallic tubes, some areas in the scene may exceed the dynamic range of camera sensor due to problems such as reflections, shadows and uneven lighting, which also means over-saturated or under-saturated regions may appear in tube images. One single low dynamic range (LDR) image cannot provide complete and absolute tube contour information in the process of detection;
- Low contour label accuracy. It is difficult to accurately label the tube contours by manual, and the binary labels naturally have jagged morphology instead of ideal smooth contours. These factors will directly affect the performance of networks.

### 1.2. Contributions

In order to improve the accuracy and integrity of tube contour detection under complex background, we present a novel FCN-based tube contour detection method. The main contributions of this paper are as follows:

- We propose to use high-resolution multi-exposure (ME) images as the input of an FCN model. These ME images of different dynamic ranges can guarantee the integrity of tube contour information;
- A new loss function, which is calculated based on dilated contours, is introduced and used in the training of FCN. Minimizing this loss function will help to eliminate the adverse effects caused by positional deviation and jagged morphology of tube contour labels;
- We present a new dataset and a new evaluation metric to verify the effectiveness of our proposed method in the tube contour detection task.

## 2. Method

Currently, most of the neural networks used for image segmentation and edge detection have the following three characteristics: (1) FCN architecture is adopted to ensure that they can be trained end-to-end and provide efficient dense prediction; (2) deconvolutional layers are used to restore the condensed feature maps to full-size at the top of these networks; (3) skip connections are used for combining semantic information from deep layers with appearance information from shallow layers to produce accurate pixel-level classification. As one typical implementation of FCN, the U-Net [12] adopts an encoder-decoder architecture, which is not only simple in structure but also has been proven to be an effective model for image segmentation tasks with small datasets [15,16]. The FCN used for tube contour detection in this study is modified based on the U-Net. What follows is a detailed description of the network architecture and the loss function adopted in the training process.

### 2.1. Network Architecture

**Figure 2:**
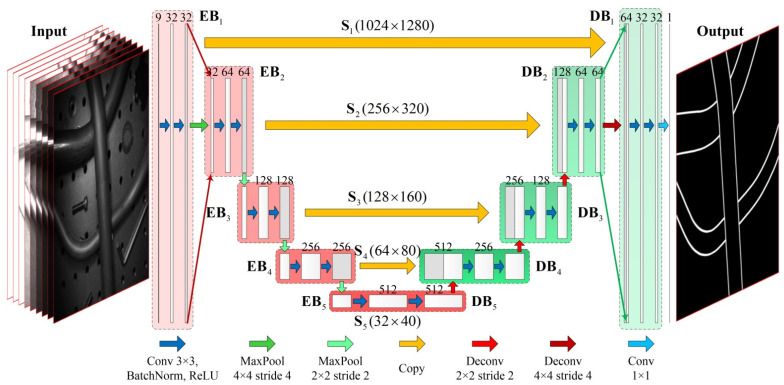
The architecture of the designed FCN.

The architecture of the FCN is shown in Figure 2. In this figure, the rectangular blocks represent the feature maps. The height of each rectangular block reflects the size of the feature map, and the width of each rectangular block represents the number of channels in the feature map. In addition, the arrows between the feature maps (except for the orange arrows) represent the layer operations. Different colored arrows indicate different kinds of layers. The detailed correspondence is illustrated at the bottom of the figure. The orange arrows in the figure indicate copying of feature maps.

The FCN takes ME images of a static scene instead of one single image as input. Each group includes under-exposure, normal-exposure, and over-exposure images, to ensure that the network can obtain the information of tube contours in different dynamic ranges. Here, we take 9 images with resolution of 1024 × 1248 pixels as an example to illustrate the architecture of the customized FCN model based on the U-Net.

The input ME images are first passed through a contracting path containing 5 encode blocks ($EB_{1}\sim EB_{5}$) to extract multi-dimensional feature maps under 5 different spatial scales ($S_{1}\sim S_{5}$). Each encode block contains two 3 × 3 convolutional layers with 1 pixel zero padding, and each convolutional layer followed by a batch normalization layer and a rectified linear unit (ReLU). The adjacent encode blocks are connected by max pooling layers for down-sampling. The number of feature channels in $EB_{1}$ is 32, and it is doubled at each down-sampling step.

Subsequently, the FCN also contains an expansive path, which is composed of 5 decode blocks ($DB_{5}\sim DB_{1}$, where $DB_{5}$ is $EB_{5}$). This path is used for up-sampling the feature maps of different spatial scales, and eventually outputting a feature map, which has the same size as the input images. The internal structure of decode blocks is the same as encode blocks, but the adjacent decode blocks are connected by deconvolutional layers for up-sampling the feature maps. The number of feature channels is halved at each up-sampling step.

In addition, there are three points to note: (1) All the input feature maps of $DB_{i}$ (i=1~4) are composed of two parts: half of the channels are from the output of $EB_{i}$ on the same spatial scale $S_{i}$, and the other half are from the output of $DB_{i+1}$ followed by a deconvolutional operation. This strategy integrates the semantic information from deep layers with appearance information from shallow layers to produce accurate and detailed tube contour detection results; (2) The max pooling layer between $EB_{1}$ and $EB_{2}$, and the deconvolutional layer between $DB_{2}$ and $DB_{1}$ are with kernel size 4 × 4 and stride 4, while the rest max pooling and deconvolutional layers are with kernel size 2 × 2 and stride 2. This design aims to increase the contraction and expansion rate of the FCN, so as to reduce the memory space occupied by high-resolution feature maps and increase the receptive field size. Compared to the original U-Net [12], this modification can reduce the memory usage of training by approximately 36%; (3) The output of $DB_{1}$ is followed by a convolutional layer with kernel size 1 × 1 for converting the 32-channels feature map to a single-channel output.

**Figure 3:**
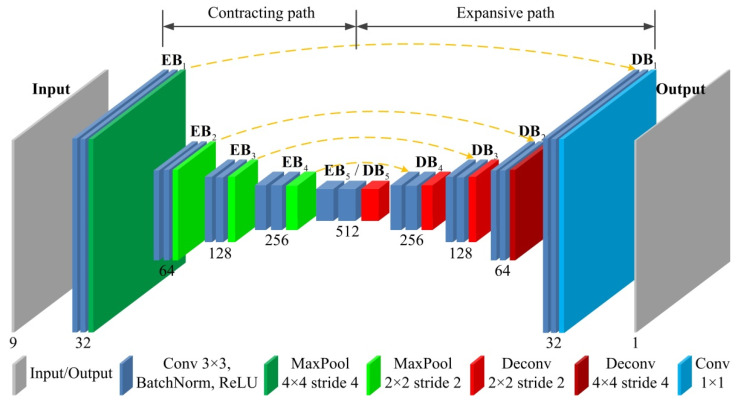
The detailed layer configuration of the designed FCN.

Based on the above descriptions, the detailed layer configuration of the designed FCN is shown in Figure 3. In this figure, rectangles with different colors indicate different kinds of layers. In summary, the proposed network contains a total of 19 convolutional layers, 4 max pooling layers and 4 deconvolutional layers, among which the first 18 convolutional layers adopt kernel size 3 × 3, with each followed by a batch normalization layer and a ReLU layer, while the kernel size of the last convolutional layer is 1 × 1. Besides, the first max pooling layer in the contracting path and the last deconvolutional layer in the expansive path are both with kernel size 4 × 4 and stride 4, while the rest max pooling layers and deconvolutional layers are both with kernel size 2 × 2 and stride 2. In addition, the dimensions of the input and output feature maps of each block in the designed FCN are shown in Table 1.

### 2.2. Loss Function

**Figure 4:**
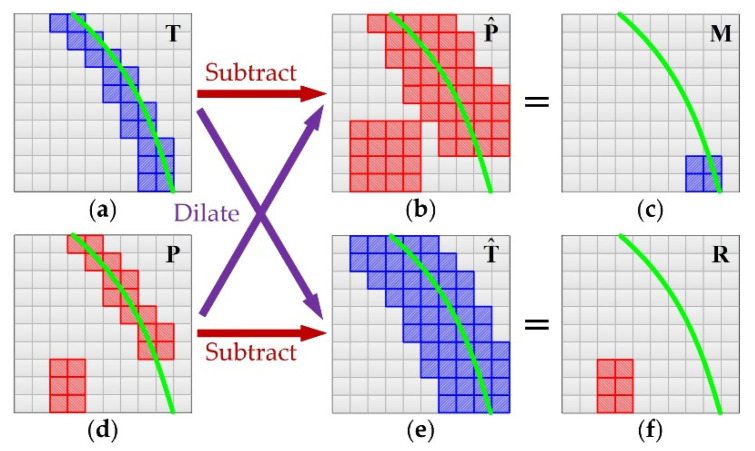
The calculation process of DC loss. (**a**) Ground truth; (**b**) dilated probability map; (**c**) missing contour; (**d**) probability map; (**e**) dilated ground truth; (**f**) redundant contour.

The FCN converts the tube contour detection task to a single-label binary classification problem. Hence, the well-known binary cross-entropy (BCE) can be used as the loss function here. Let the input ME images be $\left\{I_{1},\ldots ,I_{H}\right\}$ with the same size of M×N. O denotes the single-channel output of the FCN, and T denotes the ground truth contour label corresponding to the input data, where the essence of T is a binary image of the real contour (0 indicates non-contour, and 1 indicates contour). The output O first needs to be converted to the tube contour’s probability map P by sigmoid function, and then the BCE loss can be expressed as

(1) $${ℒ}_{BCE}=-\sum_{i=0}^{M}\sum_{j=0}^{N}w_{T_{ij}}\left[T_{ij}\log(P_{ij})+\left(1-T_{ij}\right)\log(1-P_{ij})\right]$$

where $P_{ij}=1/\left(1+\exp^{-O_{ij}}\right)$, and i and j represent the row number and column number respectively. Besides, considering that there are fewer pixels on the tube contours, we also introduce the weight coefficient $w_{T_{ij}}$ into the loss function to balance the positive and negative samples. The implicit premise of using BCE loss is that the contour labels are absolutely accurate. Otherwise, the FCN will be forced to learn inaccurate contours, which is directly manifested as overfitting. However, there are inevitably positional deviations between the manually labeled tube contours and the real tube contours. Meanwhile, the binary labels naturally have jagged morphology. These two factors are not conducive to the training of FCN. Therefore, we propose a new loss function, called dilated contour (DC) loss, for the FCN training. The specific calculation process of DC loss is shown in Figure 4.

Similar to BCE loss, the calculation process of DC loss also depends on the tube contour’s label T and probability map P, as shown in Figure 4a,d. The blue boxes in Figure 4a indicate pixels labeled as tube contour, the red boxes in Figure 4d indicate pixels predicted as tube contour and the green curve indicates the real position of tube contour. First, we perform a morphological dilation operation on T and P, respectively, so as to expand the contour width. This process is the same as max pooling, which slides a window of fixed size (3 × 3) over the input and takes the max value in the window. Then, we get the dilated contours as shown in Figure 4b,e. The process of dilation can be expressed as follows:

(2) $$\{\begin{array}{c} {\hat{P}}_{ij}=\underset{i^{\prime },j^{\prime }\in \left\{-1,0,1\right\}}{\max}P\left(i+i^{\prime },j+j^{\prime }\right)\\ {\hat{T}}_{ij}=\underset{i^{\prime },j^{\prime }\in \left\{-1,0,1\right\}}{\max}T\left(i+i^{\prime },j+j^{\prime }\right) \end{array}$$

Then, we subtract $\hat{P}$ from T and $\hat{T}$ from P. This process can be expressed as follows:

(3) $$\{\begin{array}{c} M_{ij}=\underset{\left(0,1\right)}{\mathrm{sat}}\;\left(T_{ij}-{\hat{P}}_{ij}\right)\\ R_{ij}=\underset{\left(0,1\right)}{\mathrm{sat}}\;\left(P_{ij}-{\hat{T}}_{ij}\right) \end{array}$$

where sat denotes saturation arithmetic that limits the result to a fixed range, which is 0~1. The subtraction results M and R represent the missing and redundant contours of P, respectively, as shown in Figure 4c,f. Finally, the DC loss can be calculated by M, R and T, as follows:

(4) $${ℒ}_{DC}=\frac{\sum_{i=0}^{M}\sum_{j=0}^{N}\left(M_{ij}+R_{ij}\right)}{\sum_{i=0}^{M}\sum_{j=0}^{N}T_{ij}}$$

The DC loss mainly reflects the missing contours and redundant contours due to the introduction of dilation operation. Therefore, the use of DC loss can effectively eliminate the adverse effects caused by positional deviation and jagged morphology of tube contour labels. However, the basic premise of using DC loss is that the FCN can output approximate binary probability maps, which is difficult to meet in the early stage of training. Therefore, its necessary to comprehensively use BCE loss and DC loss in the whole training process. The effectiveness of this training strategy will be verified in Section 4.2.2.

## 3. Multi-Exposure Tube Contour Dataset

Considering that there is no appropriate public dataset available for the overall evaluation of our proposed method, we introduce and share a new dataset called multi-exposure tube contour dataset (METCD). Each sample of this dataset contains ME images of a static scene constructed with tubes, the corresponding high dynamic range (HDR) image and tube contour labels with different widths.

### 3.1. Multi-Exposure Image Acquisition

**Figure 5:**
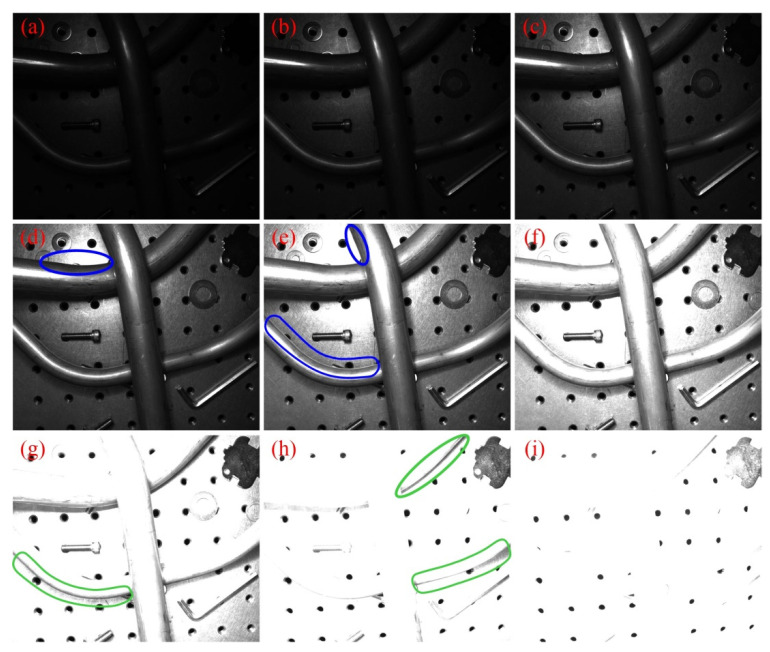
A sequence of tube ME images with different exposure times. (**a**) 2048 μs; (**b**) 4096 μs; (**c**) 8192 μs; (**d**) 16,384 μs; (**e**) 32,768 μs; (**f**) 65,536 μs; (**g**) 131,072 μs; (**h**) 262,144 μs; and (**i**) 524,288 μs.

We constructed a ME images acquisition system, which consisted of an industrial camera (MER-504-10GM-P) from Daheng, an industrial lens (M1214-MP2) from Computar and a LED ring light source. The lens aperture was set to f/5.6. The acquisition objects were 7 metallic tubes; 4 of them were used to acquire train set, while the other 3 were used to acquire validation set. Besides, all these tubes were used to acquire test set. For each scene, we randomly placed 1~3 tubes and some distraction objects (such as bolts, nuts, and washers) in the camera’s field of view, and then photographed with 9 different exposure times (2048, 4096, 8192, 16,384, 32,768, 65,536, 131,072, 262,144 and 524,288 μs). The acquired ME images were resize to 1024 × 1280 pixels at last. A sequence of captured tube ME images is shown in Figure 5.

As we can see from Figure 5, the parameter settings described above can ensure that each group of ME images includes under-exposure, normal-exposure and over-exposure images. By comparison, it can be found that the blue areas in Figure 5d,e tend to reflect light, so the tube contours can be better represented in the images with shorter exposure times. While the green areas in Figure 5g,h are on the dark side, the tube contours can be better represented in the images with longer exposure times. This proves that the use of ME images can effectively guarantee the information integrity of the tube contours. The METCD contains ME images of 72 different scenes, 30 of them are used for FCN training (train set), 10 of them are used for evaluation (validation set) and the rest are used for additional testing (test set).

### 3.2. Labeling Process

**Figure 6:**
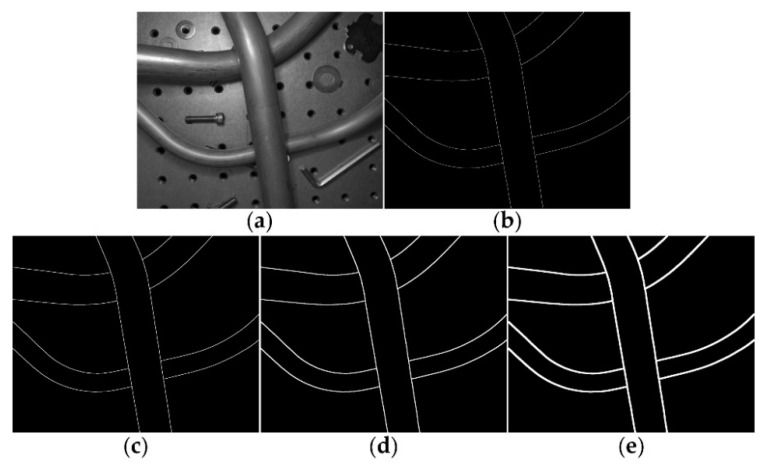
The tone mapped HDR image and the corresponding tube contour labels with different widths. (**a**) Tone mapped HDR image; (**b**) 1-pixel contour width; (**c**) 2-pixel contour width; (**d**) 4-pixel contour width; and (**e**) 8-pixel contour width.

As shown in Figure 5, due to problems such as reflections, shadows and uneven lighting, one single LDR image cannot guarantee that the tube contours all appear as obvious step edges, while an HDR image can represent a greater range of luminance levels [30], which will help to realize the precise labeling of tube contours. Therefore, first we fused the ME images into HDR images using the method proposed by Debevec [31], then we used tonemapper with bilateral filtering and set 2.2 as the value for gamma correction to make the HDR images displayable and finally the tube contours were labeled based on these displayable HDR images. However, it is inevitable that there will be positional deviations (about 1~2 pixels) between the manually labeled tube contours and the real tube contours. We provided four labels with different tube contour widths (1, 2, 4 and 8 pixels) for each sample of METCD. The influence of contour width on the performance of the proposed FCN will be discussed in Section 4.2.3. The tone mapped HDR image of Figure 5 and the corresponding tube contour labels with different widths are shown in Figure 6.

### 3.3. Evaluation Metrics

Tube contour detection is essentially binary classification for each pixel. Therefore, the classical metrics average precision (AP) and maximum F-measure at optimal dataset scale (MF-ODS) are employed to evaluate the performance of different methods on METCD. Here, the optimal dataset scale (ODS) refers to the best result at the optimal threshold across the entire validation set. We also notice that the number of contour pixels is significantly smaller than the number of non-contour pixels. In consequence, the maximum Matthews correlation coefficient at optimal dataset scale (MCC-ODS), which is less influenced by imbalanced data [32], is adopted as another evaluation metric.

**Figure 7:**
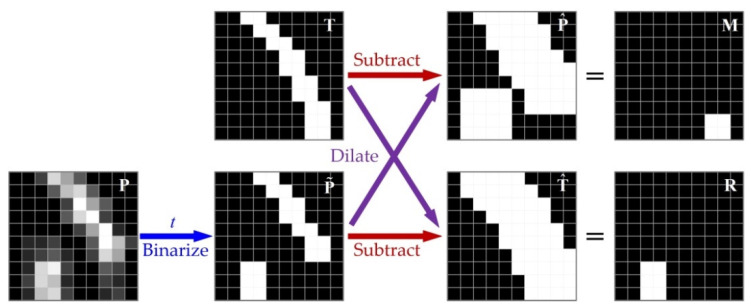
The calculation process of DIA for a single sample.

In addition, for the same reason as using DC loss, we propose a new evaluation metric called dilate inaccuracy at optimal dataset scale (DIA-ODS) to evaluate the performance of FCN in the tube contour detection task. Figure 7 shows the calculation process of dilate inaccuracy (DIA) for a single sample. In this figure, each small square represents a pixel (black indicates non-contour pixels and white indicates contour pixels). As can be seen from this figure, the calculation process of DIA is quite similar to that of the DC loss shown in Figure 4. The main difference between these two processes is that the calculation of DIA requires a threshold t∈[0,1] to perform binarization processing on the probability map P. This process can be expressed as follows:

(5) $${\tilde{P}}_{ij}=\{\begin{array}{c} 1\\ 0 \end{array}\;\begin{array}{l} P_{ij}>t\\ \mathrm{otherwise} \end{array}$$

Then, we substituted the binarized probability map $\tilde{P}$ and the ground truth label T into Equation (2) (dilate operation) and Equation (3) (subtract operation), to obtain the missing contour M and redundant contour R. Finally, the ratio of the sum number of white pixels in M and R to the total number of the white pixels in T is the DIA of the sample under the current threshold t. Let us take the situation shown in Figure 7 as an example, there are 20 white pixels in T, and there are 4 and 6 white pixels in M and R respectively. Therefore, the DIA of this sample calculated under the current threshold is (4+6)/20=0.5.

The above DIA calculation process is completed for a single sample. Suppose there are K samples in the validation set, then there will be probability maps $\left\{P_{1},\ldots ,P_{K}\right\}$ and ground truth labels $\left\{T_{1},\ldots ,T_{K}\right\}$. We choose a fixed threshold t to perform binarization processing on all the $\left\{P_{1},\ldots ,P_{K}\right\}$ and get the binarized probability maps $\left\{{\tilde{P}}_{1},\ldots ,{\tilde{P}}_{K}\right\}$. Then, the calculation of the missing contours $\left\{M_{1},\ldots ,M_{K}\right\}$ and redundant contours $\left\{R_{1},\ldots ,R_{K}\right\}$ for all samples are completed based on the method described in Figure 7. Finally, the DIA calculated on the validation set can be expressed as:

(6) $$DIA=\frac{\sum_{k=1}^{K}\sum_{i=0}^{M}\sum_{j=0}^{N}\left(M_{ijk}+R_{ijk}\right)}{\sum_{k=1}^{K}\sum_{i=0}^{M}\sum_{j=0}^{N}T_{ijk}}$$

It is not difficult to find from the above description that different threshold values will yield different DIA results. We select the minimum DIA across the entire validation set by varying threshold t, then we get DIA-ODS. Obviously, the lower DIA-ODS indicates the better performance.

## 4. Experiments

In order to verify the effectiveness of the proposed method, we conducted several experiments on the METCD. First the hyper-parameters and training strategies adopted in this paper will be detailed in Section 4.1, and then comparative experiments from four aspects are given in Section 4.2. The source code and METCD are available in the GitHub repository: https://github.com/chexqi/Tube_Contour_Detection, accessed on 24 April 2021.

### 4.1. Experimental Framework

- Data Augmentation: Due to the small number of training samples in the METCD, data augmentation was performed to improve the generalization ability of the network and avoid overfitting. We augmented the training data with the following ways: (1) random horizontal and vertical flipping with probability 0.5; (2) random rotation between (−45°, +45°); (3) random horizontal and vertical translation in the scale of (−0.2, +0.2); and (4) random scaling in the range of (1, 1.2).
- Optimization: The Adam optimizer was used as the optimization algorithm. The size of mini-batches was set to 3. The learning rate was initialized to 0.01 and decayed by a factor of 0.9 every 70 epochs. We ran all experiments for 700 epochs. The joint loss function adopted in the training process was combined by BCE loss and DC loss, as follows:

(7) $$ℒ=w_{BCE}{ℒ}_{BCE}+w_{DC}{ℒ}_{DC}$$

where $w_{BCE},w_{DC}\in \left\{0,1\right\}$, and they are indicate whether ${ℒ}_{BCE}$ and ${ℒ}_{DC}$ were used in the calculation process of ℒ, respectively. In addition, in order to solve the problem of imbalance between positive and negative samples, the weight coefficient in Equation (1) was assigned as

(8) $$w_{T_{ij}}=\{\begin{array}{c} 1\;if\;T_{ij}=0\\ 2\;if\;T_{ij}=1 \end{array}$$

- Implementation: All experiments were conducted on a machine equipped with an Intel Core i9-9900X CPU, 32GB RAM and a NVIDIA GTX 2080Ti GPU.

### 4.2. Experimental Results

First, we conducted experiments from two aspects, data input and loss function, to validate the effectiveness of using ME images and DC loss. Then, we evaluated the influence of contour width on the performance of the FCN model. Finally, our proposed method was compared with other neural network-based methods. We evaluated each model using the metrics illustrated in Section 3.3 (AP, MF-ODS, MCC-ODS and DIA-ODS), and all these metrics are shown by % in this paper.

#### 4.2.1. Data Input

**Figure 8:**
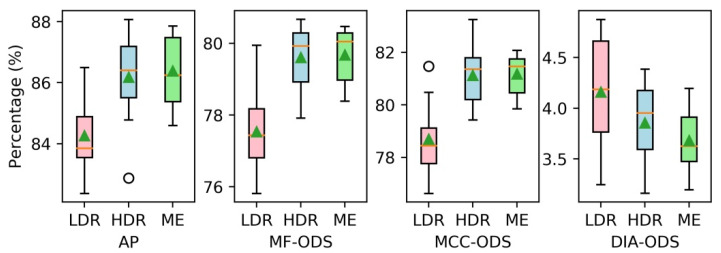
The performances of FCN models with different inputs.

In order to prove the validity of using ME images as input, we compared the performance of our proposed FCN with three different inputs: one with a single normally exposed LDR image (LDR-FCN), one with a single fused HDR image (HDR-FCN) and one with a sequence of ME images (ME-FCN). In this experiment, the contour labels with 2 pixels width were adopted for training, and the network was optimized only by minimizing the traditional BCE loss ($w_{BCE}=1$, $w_{DC}=0$). The other parameters followed the settings described in Section 4.1. In addition, considering the uncertainty caused by random initialization and data augmentation, we ran each test 10 times and averaged the results. The performances of FCN models with different inputs on the validation set are presented in Figure 8 using the box diagram method, while the mean values of evaluation metrics are reported in Table 2.

**Figure 9:**
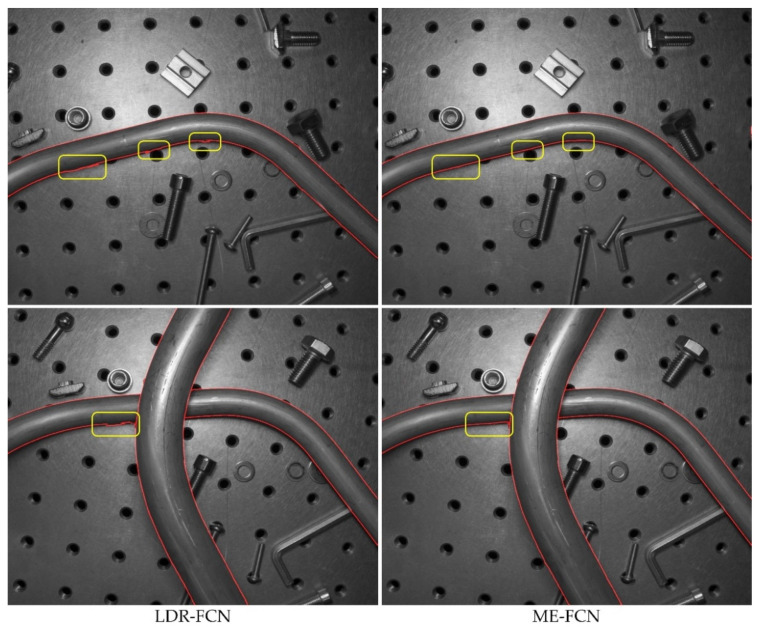
Tube contour detection results on several samples. The images in the left column were produced by an FCN model that took the LDR image as input, and in the right column were produced by an FCN model that took ME images as input.

It can be clearly observed that the HDR-FCN models and the ME-FCN models achieve higher AP, MF-ODS and MCC-ODS and lower DIA-ODS than the LDR-FCN models. Furthermore, the ME-FCN models give the best performance in term of AP (86.4%), MF-ODS (79.7%), MCC-ODS (81.2%) and DIA-ODS (3.7%), and avoid using the HDR image fusion operation. Figure 9 shows some comparative tube contour detection results between an LDR-FCN model and an ME-FCN model.

#### 4.2.2. Loss Function

**Figure 10:**
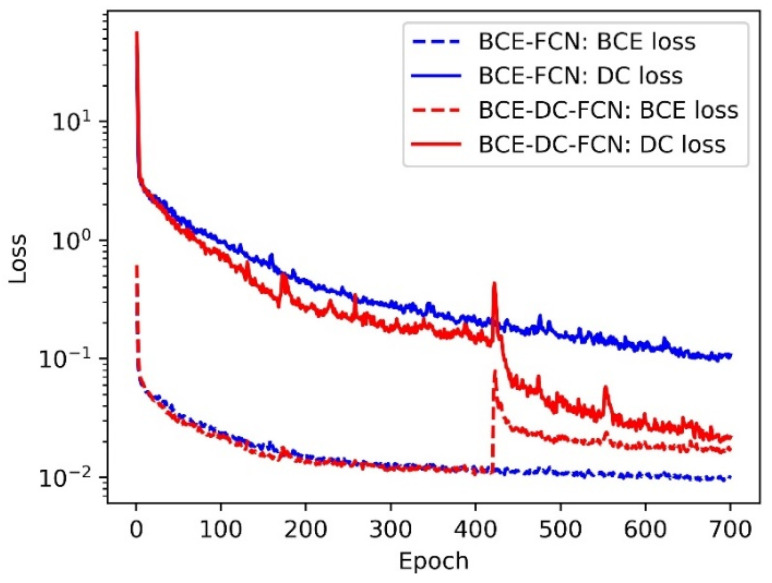
The BCE loss and DC loss of the training processes for FCN models trained with different losses.

On the basis of verifying the effectiveness of taking ME images as input, experiment about loss function was conducted. The FCN models in this experiment were trained with two different losses: one with BCE loss only (BCE-FCN, $w_{BCE}=1$, $w_{DC}=0$), and the other with both BCE loss and DC loss (BCE-DC-FCN, $w_{BCE}=1$, $w_{DC}=0$ in the first 420 epochs and $w_{BCE}=1$, $w_{DC}=1$ in the following 280 epochs). The contour labels with 2 pixels width were also adopted here for training. Figure 10 shows the BCE loss and DC loss of the training processes for different FCN models.

**Figure 11:**
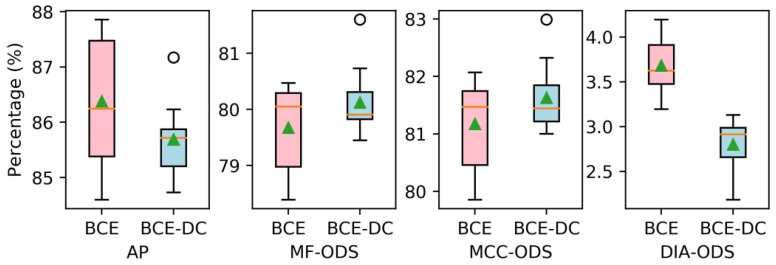
The performances of FCN models trained with different losses.

The blue curves and the red curves indicate the losses of BCE-FCN model and BCE-DC-FCN model, respectively, while the dashed curves and solid curves refer to the values of BCE loss and DC loss, respectively. As can be seen from Figure 10, compared with FCN trained with only BCE loss, although the training process of BCE-DC-FCN achieves higher BCE loss, it can also effectively reduce DC loss. Then, we ran each test 10 times. The performances of FCN models trained with different loss functions are presented in Figure 11, while the mean values of evaluation metrics are reported in Table 3.

**Figure 12:**
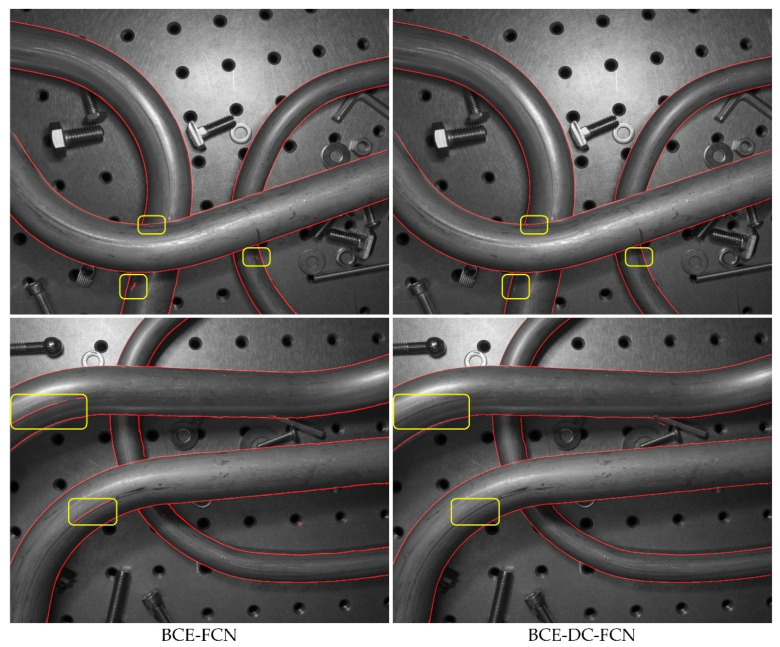
Tube contour detection results on several samples. The images in the left column were produced by an FCN model that was trained with BCE loss, and in the right column were produced by an FCN model that was trained with BCE-DC loss.

As can be seen from Figure 11 and Table 3, the BCE-DC-FCN models almost achieve the same performance in terms of AP (86.4% to 85.7%), MF-ODS (79.7% to 80.1%) and MCC-ODS (81.2% to 81.6%) as the BCE-FCN models, but they significantly reduce the DIA-ODS (3.7% to 2.8%). This indicates that the tube contours detected by BCE-DC-FCN models are completer and more correct (fewer missing contours and redundant contours). Figure 12 shows some comparative tube contour detection results between a BCE-FCN model and a BCE-DC-FCN model.

#### 4.2.3. Contour Width

**Figure 13:**
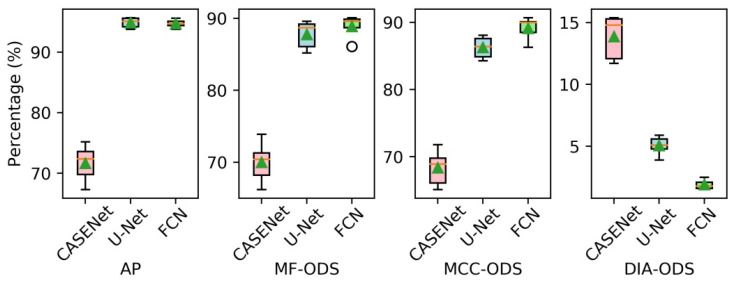
The performances of FCN models trained on labels with different contour widths.

Due to the positional deviation and jagged morphology of contour labels, its impossible to achieve absolutely accurate labeling of tube contours. When labels with different contour widths are used for the FCN training and evaluation, the proportion and effect of mislabeled pixels will be different. Therefore, on the basis of confirming the effectiveness of using ME images and BCE-DC loss, we tested the influence of labels with different contour widths on the performance of FCN. The FCN models in this experiment were divided into four groups. They were trained on labels with 1-pixel (Width1-FCN), 2-pixel (Width2-FCN), 4-pixel (Width4-FCN) and 8-pixel (Width8-FCN) contour width, respectively. The other parameters followed the settings described in Section 4.1. The box diagrams and mean values of obtained AP, MF-ODS, MCC-ODS and DIA-ODS are shown in Figure 13 and Table 4, respectively, which depict results after 10 evaluations.

The experimental results show that the AP, MF-ODS and MCC-ODS increase significantly with the increase of contour width. This is mainly due to the decreasing proportion of mislabeled pixels in tube contour labels. However, it should be noted that the DIA-ODS is not effectively reduced when contour width is larger than 2 pixels, which means the missing and redundant contours are not effectively reduced, while the width of the predicted tube contours will be wider as the contour width of ground truth labels increases. This phenomenon is unfavorable for three-dimensional reconstruction and measurement of tubes.

#### 4.2.4. Comparison with Other Methods

**Figure 14:**
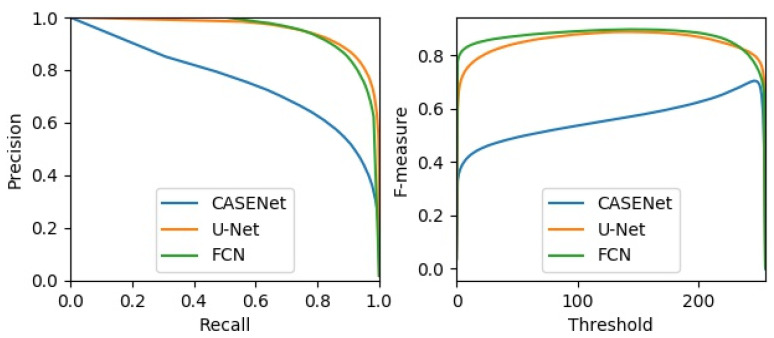
Comparison of precision-recall curves and F-measure curves of different methods.

**Figure 15:**
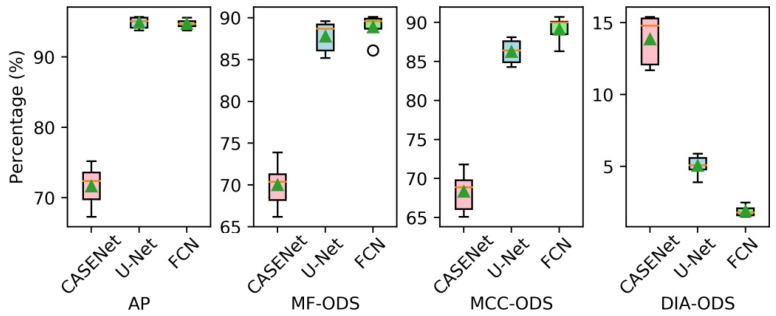
The performances of different methods.

In the following, we compared our method with other neural network-based methods that can be used for tube contour detection. The experiment was divided into three groups. In the first group, the CASENet [24] was employed for tube contour detection. This network has been proved to be effective for large-scale semantic edge detection tasks. In the second group, the original U-Net [12] was used for tube contour detection. This network has achieved good results in various image segmentation tasks with small datasets. Our FCN was adopted for tube contour detection in the third group. All the three groups in the experiment were trained and tested on the METCD. Considering that if the high-resolution images (1024 × 1280 pixels) were directly used as the input of CASENet and original U-Net, the memory used in training with a mini-batch of 3 would reach up to 24.3 GB and 33.9 GB respectively. This was beyond the hardware limit of the experimental platform. So, we first resized the tube images and the 4 pixel-width labels to 512 × 640 pixels and then carried out experiments with these low-resolution samples. The data enhancement method described in Section 4.1 was adopted by all networks. In addition, the training process of CASENet adopted the multi-label loss described in [24], while the training process of the original U-Net adopted the dice loss introduced in [33]. Figure 14 shows the precision-recall curves and the F-measure curves of different methods. The box diagrams and mean values of obtained AP, MF-ODS, MCC-ODS and DIA-ODS are shown in Figure 15 and Table 5, respectively, which depict results after 10 evaluations.

**Figure 16:**
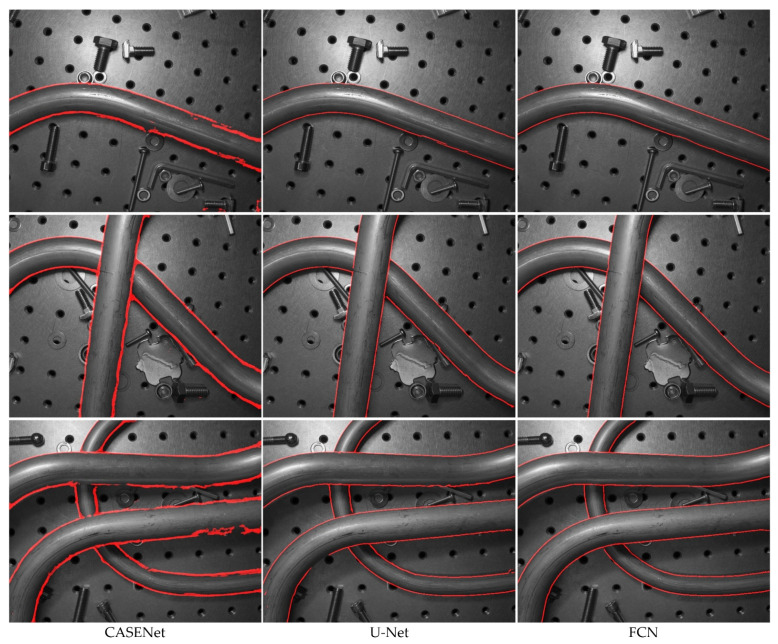
Tube contour detection results of comparing methods on the METCD. The images in the left column were produced by the CASENet model, in the middle column were produced by the original U-Net model and in the right column were produced by our FCN model.

As can be seen from Figure 14, Figure 15 and Table 5, the CASENet models achieve the worst performance in terms of AP (71.7%), MF-ODS (70.0%), MCC-ODS (68.3%) and DIA-ODS (13.9%). This indicates that the CASENet is not applicable to the tube contour detection task with small dataset. The original U-Net models and our FCN models almost achieve the same performance in terms of AP (94.9% and 94.7%), MF-ODS (87.8% and 88.9%) and MCC-ODS (86.3% and 89.1%), but our FCN models achieve lower DIA-ODS (1.9%). Figure 16 shows some comparative tube contour detection results between the three kinds of methods.

As can be seen from the above experimental results, although our FCN architecture accelerates the contraction and expansion rate of feature maps and enables high-resolution images to be used as input, this does not reduce the performance of tube contour detection. In addition, benefits from the using of ME images and BCE-DC loss are that the tube contours detected by our FCN model are completer and more correct.

**Figure 17:**
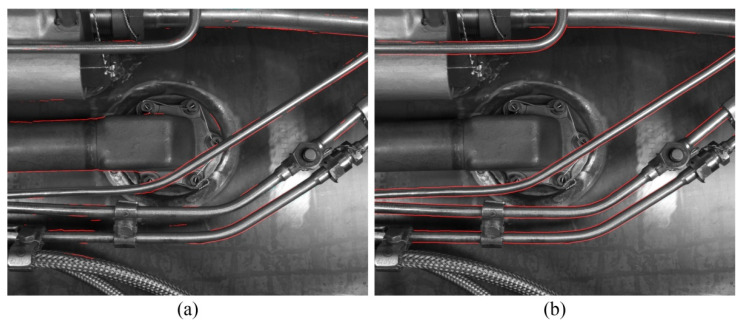
The detection effect of tube contours under a new scenario. (**a**) Using an FCN model trained on the METCD; (**b**) using an FCN model retrained on the new labeled dataset.

Its important to note that the METCD presented in this paper is a small dataset with only 30 samples for training. Therefore, the FCN model trained on the METCD is not applicable to other new scenarios. Figure 17 shows the detection effect of tube contours under a new scenario. Among them, Figure 17a presents the result obtained by an FCN model trained on the METCD and Figure 17b presents the result obtained by an FCN model retrained on the new labeled dataset. It can be seen that under the new scenario, it is necessary to label a new dataset and retrain the model to get the best performance.

To the best of our knowledge, this is the first paper in which deep learning method is employed in the tube contour detection task under complex background. The experiments in this section also proved that the proposed method is more suitable for the task of tube contour detection than the other existing neural networks that can be used for the same task. Moreover, the METCD introduced and shared in this paper also provides a useful benchmark for the community of tube contour detection methods. We believe that such a finely labeled dataset is very important for the training and evaluation of new developed tube contour detection methods.

## 5. Conclusions

Aiming at the practical problems encountered in the application of tube contour detection under complex background, we propose a new FCN-based tube contour detection method. Our FCN architecture is modified based on the U-Net. We accelerate the contraction and expansion rate of feature maps, so as to enable the FCN model to take high-resolution images as input and produce a thin tube contour detection result. In order to solve the problem of poor tube image quality, ME images of a static scene are used as the input of the FCN model. In addition, we propose a new loss function, namely, DC loss, to solve the problem of low contour label accuracy. We also present a new dataset called METCD and a new evaluation metric called DIA-ODS to evaluate the performance of the proposed method. The experimental results show that the proposed method can effectively improve the integrity and accuracy of tube contour detection in complex scenes.

## Figures and Tables

**Table 1:** The dimensions of feature maps in different spatial scales.

Spatial Scale	Contracting Path			Expansive Path		
	Block	Input	Output	Block	Input	Output
$S_{1}$	$EB_{1}$	1028 × 1280 × 9	1028 × 1280 × 32	$DB_{1}$	1028 × 1280 × 64	1028 × 1280 × 32
$S_{2}$	$EB_{2}$	256 × 320 × 32	256 × 320 × 64	$DB_{2}$	256 × 320 × 128	256 × 320 × 64
$S_{3}$	$EB_{3}$	128 × 160 × 64	128 × 160 × 128	$DB_{3}$	128 × 160 × 256	128 × 160 × 128
$S_{4}$	$EB_{4}$	64 × 80 × 128	64 × 80 × 256	$DB_{4}$	64 × 80 × 512	64 × 80 × 256
$S_{5}$	Block		Input		Output	
	$EB_{5}\left(DB_{5}\right)$		32 × 40 × 256		32 × 40 × 512	

**Table 2:** The mean values of evaluation metrics computed from FCN models with different inputs.

Models	AP (%)	MF-ODS (%)	MCC-ODS (%)	DIA-ODS (%)
LDR-FCN	84.3	77.5	78.7	4.2
HDR-FCN	86.2	79.6	81.1	3.9
ME-FCN	86.4	79.7	81.2	3.7

**Table 3:** The mean values of evaluation metrics computed from FCN models trained with different losses.

Models	AP (%)	MF-ODS (%)	MCC-ODS (%)	DIA-ODS (%)
BCE-FCN	86.4	79.7	81.2	3.7
BCE-DC-FCN	85.7	80.1	81.6	2.8

**Table 4:** The mean values of evaluation metrics computed from FCN models trained on labels with different contour widths.

Models	AP (%)	MF-ODS (%)	MCC-ODS (%)	DIA-ODS (%)
Width1-FCN	58.6	62.7	66.3	4.9
Width2-FCN	85.7	80.1	81.6	2.8
Width4-FCN	94.8	87.7	87.8	2.5
Width8-FCN	98.0	92.2	91.5	2.6

**Table 5:** The mean values of evaluation metrics computed from different methods.

Models	AP (%)	MF-ODS (%)	MCC-ODS (%)	DIA-ODS (%)
CASENet	71.7	70.0	68.3	13.9
U-Net	94.9	87.8	86.3	5.1
FCN	94.7	88.9	89.1	1.9

## Data Availability

The source code and METCD are available in the GitHub repository (https://github.com/chexqi/Tube_Contour_Detection), accessed on 24 April 2021.
